# Genome sequence of the New Zealand raw milk isolate and candidate probiotic strain *Lactococcus lactis* FNZ339

**DOI:** 10.1128/mra.00648-24

**Published:** 2024-09-30

**Authors:** Eduardo L. de Almeida, Paul W. O’Toole, Shalome A. Bassett

**Affiliations:** 1 Fonterra Microbiome Research Centre, University College Cork, Cork, Ireland; 2 APC Microbiome Ireland, University College Cork, Cork, Ireland; 3 School of Microbiology, University College Cork, Cork, Ireland; 4 Fonterra Research and Development Centre, Palmerston North, New Zealand; 5 Riddet Institute, Massey University, Palmerston North, New Zealand; The University of Arizona, Tucson, Arizona, USA

**Keywords:** genomics, bioinformatics, probiotics, *Lactococcus lactis* FNZ339

## Abstract

The complete genome sequence of the candidate probiotic strain *Lactococcus lactis* FNZ339 was determined using a hybrid genome assembly comprising data from Illumina and Oxford Nanopore Technologies sequencing platforms. The genome assembly comprised 2,673,297 bp, including the complete circular chromosome and four circular plasmids.

## ANNOUNCEMENT

*Lactococcus lactis* FNZ339 was obtained from the Fonterra Culture Collection (Palmerston North, New Zealand) and deposited with the Australian Measurement Institute under deposit number V23/018421. The strain was isolated from raw bovine milk on M17 Agar (30°C, 48 h) and purity streaked twice. FNZ339 was originally assigned as a *Leuconostoc* sp. using traditional culture methods. However, the final taxonomy, as determined by this study, was *L. lactis*.

We used a hybrid genome assembly approach combining Illumina and Oxford Nanopore Technologies (ONT) reads. The strain was purity streaked twice from the original −80°C glycerol stock. A single colony was incubated statically overnight (M17 broth, 22°C) for master and working glycerol stocks, and gDNA isolation for Illumina sequencing. DNA extraction for ONT sequencing required an additional culturing step where 1 mL overnight culture was added to 12 mL pre-warmed M17 broth and incubated at 22°C for 5 h. For Illumina sequencing, >200 ng of total gDNA was extracted using the QIAamp DNA Mini Kit (Qiagen, Germany), a library was prepared using the Nextera XT DNA Library Preparation Kit (Illumina, USA), and sequencing was performed on the Illumina MiSeq platform. Paired 150 bp reads (3,003,319 total reads) were processed using fastQC v0.11.9 (1) and Trim Galore v0.6.7 (2), adapters removed and read ends below Q30 trimmed. For ONT GridION sequencing, >240 ng of high molecular weight gDNA was extracted using the NucleoBond HMW DNA Kit (Macherey-Nagel, Germany). A library was prepared using the ONT Rapid Barcoding Kit (ONT, UK) and run on R10.4.1 flow cells. Base-calling was done using Guppy v4.3.4, resulting in 159,822 raw reads and *N*_50_ = 13,142 bp after removing reads with *Q* < 9.

Unicycler v0.4.8 (3) was employed for genome assembly, contig circularity determination, and rotation. Assembly statistics were computed using QUAST v5.2.0 (4), SeqKit v2.5.1 (5), and Bakta v1.9.3 (6). The final genome consisted of a total length of 2,673,297 bp, *N*_50_ of 2,490,303 bp, *L*_50_ of 1, G + C content of 35.11%, with 2,678 predicted genes and an estimated 737-fold genome coverage. This included the complete circular chromosome (2,490,303 bp), four complete circular plasmids: pFNZ339-01 (65,217 bp), pFNZ339-02 (56,058 bp), pFNZ339-03 (48,406 bp), pFNZ339-04 (11,550 bp), and an unresolved contig (1,763 bp). Taxonomy classification of *L. lactis* FNZ339 was determined using GTDB-Tk v2.3.2 (7). Default parameters were used for all software unless otherwise specified.

No antimicrobial resistance or virulence genes were detected as defined by European Food Safety Authority (EFSA) recommendations (8) using AMRFinderPlus v3.12.8 (9) and Resfinder v4.1.0 (10) at 80% sequence identity and 70% coverage of the reference sequence. The presence of genes involved in biogenic amine synthesis was investigated using KofamScan v1.3.0 (11–14), and only the agmatine deaminase gene was detected. However, no clinically relevant level of any biogenic amine was detected when assessed *in vitro* by an independent laboratory (Eurofins). *In vitro* antibiotic resistance evaluation was performed using the ISO 10932|IDF 223:2010 standards for determining the minimal inhibitory concentration (MIC) of antibiotics in lactic acid bacteria (15) in accordance with EFSA guidelines (16). All MIC values were lower or equal to the EFSA cut-offs (Table 1A). D-/L-lactate production ratio was measured using the Megazyme D-/L-Lactic Acid (Rapid) Assay Kit (Neogen, Ireland), following the manufacturer’s instructions (Table 1B). Only the L-lactate isomer was detected, indicating no safety concern.

**TABLE 1:** *In vitro* safety assessment of *L. lactis* FNZ339: (A) MIC results and (B) D-/L-lactate production

(A) MIC results (µg/mL)—noting that ciprofloxacin is not required for testing on *L. lactis* strains (as per EFSA guidelines)									
	Ampicillin	Vancomycin	Gentamycin	Kanamycin	Streptomycin	Erythromycin	Clindamycin	Tetracycline	Chloramphenicol
*L. lactis* FNZ339	0.5	0.5	4	16	32	<0.125	<0.125	<0.5	4
EFSA guidelines for *L. lactis*	2	4	32	64	32	1	1	4	8

(B) D-/L-lactate production						
D-lactate concentration (g/L)	L-lactate concentration (g/L)	D-/L-lactate ratio	Total lactate (g/L)	D-lactate %	L-lactate %	Growth conditions
0	6.68	0	6.68	0%	100%	De Man–Rogosa–Sharpe (MRS), aerobic, 30°C, 24 h

The high-quality genome data and associated safety data presented support the safety of FNZ339 and its assessment as a potential probiotic.

## Data Availability

This Whole Genome Shotgun project has been deposited in GenBank under the accession number GCA_040448315.1. Sequencing reads have been deposited in the Sequence Read Archive under the accession numbers SRX24875507 for the Illumina reads, and SRX24875508 for the ONT reads.
